# A case of Arrhythmogenic right ventricular cardiomyopathy without arrhythmias

**DOI:** 10.1186/1746-1596-7-67

**Published:** 2012-06-12

**Authors:** Jia Wei, Jiarong Tang, Liming Xia, Xinshan Chen, Dao Wen Wang

**Affiliations:** 1 Department of Internal Medicine, Tongji Hospital, Tongji Medical College, Huazhong University of Science and Technology, 1095 Jie-Fang Ave, Wuhan 430030, China; 2 Department of Radiology, Tongji Hospital, Tongji Medical College, Huazhong University of Science and Technology, Wuhan, 430030, China; 3 Department of Forensic Pathology, Tongji Medical College, Huazhong University of Science and Technology, Wuhan, 430030, China

**Keywords:** Arrhythmogenic, Right ventricle cardiomyopathy, Magnetic resonance imaging, Heart transplantation

## Abstract

Arrhythmogenic right ventricular cardiomyopathy (ARVC) is characterized by recurrent coma, ventricular tachycardias and the replacement of the myocardium with fatty and fibrous tissue. We described a 42-year-old female patient without clinical arrhythmias which was diagnosed as ARVC by magnetic resonance imaging (MRI), but the transvenous endomyocardial biopsy was not specific. The patient received heart transplantation due to her refractory heart failure and the pathology of explanted heart demonstrated typical replacement of fatty and fibrous tissue and piles of infiltrated lymphocytes in myocardial tissue. It is concluded that ARVC might not have any arrhythmias and inflammatory process may be involved in the mechanism of ARVC.

**Virtual slides:**

The virtual slide(s) for this article can be found here: http://www.diagnosticpathology.diagnomx.eu/vs/6573514507145351

## Background

Arrhythmogenic right ventricular cardiomyopathy (ARVC) is a heart muscle disease clinically characterized by life-threatening ventricular arrhythmias [1]. It involves progressive fibrous and fatty replacement of myocardium in the inflow, apical and outflow portions of the right ventricle, resulting in ventricular tachycardia with incurrent risk of sudden death and in a proportion of cases, progressive RV dysfunction [1-5]. The ARVC without arrhythmias has not been documented and hence has been easily misdiagnosed. Consensus diagnostic criteria were developed which included MRI, echocardiography, electrocardiography and right ventricular biopsy [6]. The right ventricular biopsy, however, often gives a false-negative result because of the segmental nature of ARVC [7,8]. In this paper, we reported a case of ARVC without arrthymias which was initially misdiagnosed as dilated cardiomyopathy but later the MRI and pathology examination of the explanted heart have proved ARVC as correct diagnosis in this atypical case. The significance of MRI and possible mechanism of ARVC were discussed.

## Case presentation

**Figure 1:**
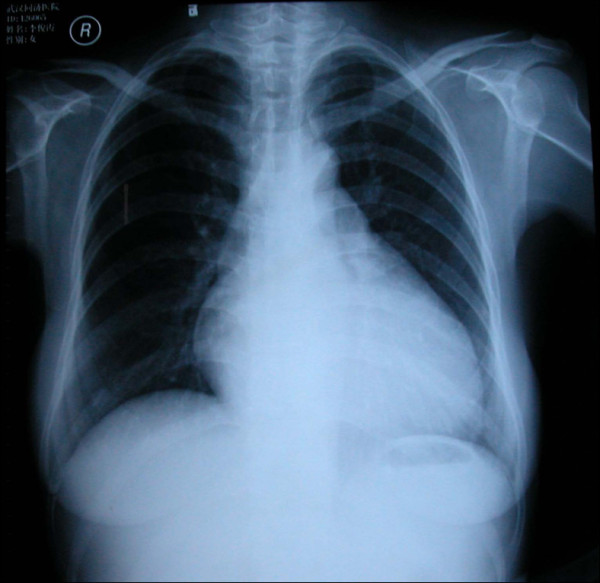
**The chest X-ray showed mild increase of transverse cardiac diameter.** The left cardiac border protruded left mildly.

**Figure 2:**
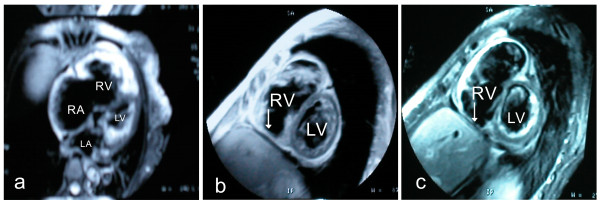
a: The cardiac MRI showed enlargement of right ventricle and atrium without enlargement of left ventricle and atrium; b: the thickness of right ventricle wall and interventricular septum was uneven; high signals indicated fatty tissue replacement (arrows); c: the signals in right ventricule free wall discontinued after inhibiting fatty tissues (arrows).

**Figure 3:**
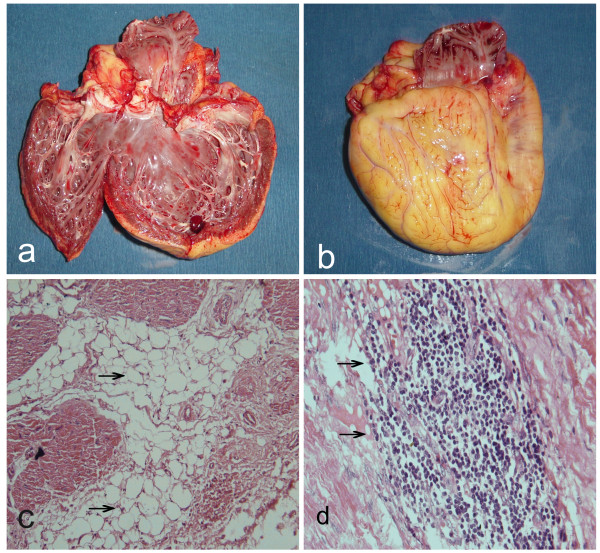
**Pathologic findings from explanted heart (gross and microscope examination).a:** the resected heart showed severe dilation of RV and thickness and fatty replacement of RV wall; **b:** the epicardium was surrounded with massive fatty tissues; **c:** the remnant cardiac muscle was located sporadically with massive fibrolipomatous tissues infiltration and myocardium was isolated like islands and structurally disordered (HE Stain × 400), arrows showing fibrolipomatous tissue replacement; **d:** massive inflammatory cells (small lymphocytes) infiltration were found in the interventricular septum under higher magnification (arrows, HE Stain × 1000).

A 42-year-old woman presented with severe abdominal distension and shortness of breath without syncope and chest pain. The patient had class IV right cardiac insufficiency by New York Heart Association (NYHA) classification. No previous similar medical history was found in her family. On physical examination, her blood pressure was 122/72 mmHg. The skin and mucosae was stained mildly yellow. She had obvious distention of jugular vein. Neither moist nor dry rale can be auscultated in her lungs. Heart rate was 98 ~ 100 bpm. 2/6 ~ 3/6 systolic cardiac murmurs can be auscultated at tricuspid valve. Her liver can be palpated 3.0 cm lower under right costal margin and 5.0 cm lower under the xiphoid process. She had positive signs of ascites with moderate to severe pitting edema at lower extremities. The total bilirubin level was 38.4umol/L. (Normal range: 3.4-20.5umol/L). The anti-myocardial antibody tests showed negative with ANT and β1 but positive with M2 and MHC. The anti-Cox-IgM and anti-CMV-IgM tests were negative but EVs-RNA was positive. Her chest X-ray showed mild increase of transverse cardiac diameter. The left cardiac border protruded left mildly (Figure 1). Electrocardiograhy demonstrated normal sinus rhythm with incomplete right bundle-branch block. Epsilon wave was apparent in leads V1 and V2. The 24 hours Holter electrocardiogram monitoring showed normal sinus rhythm with slight ST-T segment depression without any cardiac arrhythmia. In addition, the vital signs monitor did not find any visible arrhythmia during the first two weeks after admission and the preoperative stage later. The ventricular late potential test was positive in this patient. Three-dimensional echocardiography showed enlargement of right ventricle (50 mm) and right atrium (56 mm) with severe tricuspid valve insufficiency. The left ventricular ejection fraction was 32%. The right ventricle (RV) angiography showed that RV was obviously enlarged with thickness of trabecular muscles and marked hypokinesia of right ventricle myocardium with focal areas of dyskinesia. Endomyocardial biopsy shows cardiac muscle fibers were atrophied and replaced by fibrous tissues with focal small lymphocytic infiltration. The patient was then diagnosed as dilated cardiomyopathy and treated using ACE inhibitor, diuretics and digoxin, but her symptoms did not improve at all. She was then transferred into our hospital. Cardiac MRI presented the typical manifestation of ARVC which showed enlargement of right ventricle and atrium without enlargement of left ventricle and atrium (Figure 2: a); the wall of right ventricular outflow tract was thinned. In double-IR FSE, the anterior wall of right ventricle, apex and interventricular septum had high signal with fatty tissue infiltration (Figure 2: b). In triple-IR FSE, after inhibiting the fatty tissues, the signals in right ventricule free wall discontinued indicating that the myocardium in right ventricle was replaced by fibrofatty tissues (Figure 2: c). Despite maximal medical therapy, her clinical condition continued to exist and finally she underwent heart transplantation. The gross photograph of resected heart showed dilation of RV and thickness of RV wall with the weight of 300 grams, but part of biventricle wall become yellow (Figure 3: a). The epicardium was surrounded with fatty tissues (Figure 3: b). Under microscopy, the loss of myocardium was confirmed and myocardium was replaced by mature fibrofatty tissues; the remaining myocardial tissue became island-like and its structure was disordered (Figure 3: c). The remnant cardiac muscle was located sporadically in fibrofatty tissues with massive small lymphocytic infiltration (Figure 3: d). The pathological demonstration involved not only in right ventricle but left ventricle and interventricular septum as well. After heart transplantation, the patient got much better and has been still followed-up with only mild reported complaints for more than seven years.

## Discussion

ARVC is a disease pathologically characterized by fibrofatty replacement of the RV myocardium. A broad range of symptoms and signs may occur including palpitations, fatigue, syncope, chest pain and all kinds of atrial and rapid ventricular arrhythmias, especially recurrent episodes of ventricular tachycardias. Few patients presented the chronic congestive cardiac failure as chief complaint which is often confused with symptoms of mildly dilated cardiomyopathy [9]. The clinical diagnosis of ARVC without any arrhythmias is very difficult. Transvenous endomyocardial biopsy may be of great help for an in vivo morphological demonstration of fibro-fatty myocardial replacement [10], but recent studies have shown a high degree of interobserver variability in assessing fatty deposition, which may be observed even in normal hearts [1]. Endomyocardial biopsy lacks sufficient sensitivity because of the segmental nature of the disease process. Many clinicians biopsy the septum rather than the free wall of the right ventricle to avoid the risk of perforation just as this case. Correct pathological interpretation requires that a sufficient amount of myocardium be obtained without crush artifact and processed for the necessary histological, immunohistological and/or molecular diagnostic tests [11]. MRI, however, has been proved as a specific and sensitive examination technique in diagnosis of some atypical cases of ARVC [8,12]. It can detect right ventricular dilatation and aneurysm formation as well as wall-motion abnormalities including right ventricular dyskinesia; moreover, the black blood spin-echo sequences have provided excellent anatomic detail via suppressing-fat technique in our previous studies [13,14] and this technique has become the most important tool for the detection of fat and remnant myocardium in the right ventricular myocardium [8].

The etiology of ARVC is still unclear. Bowles et al found that cardiotropic viruses are more frequently identified in patients with ARVC and have been claimed to support an infective etiology of this disease [15]. But the role of these viruses in ARVC pathogenesis remains unknown. Myocardial inflammation may be seen in up to 75% of hearts at autopsy, and probably it plays a role in triggering ventricular tachyarrhythmias [16]. The pathology of explanted heart in this case showed massive focal inflammatory cells infiltration and this finding may be correlated with pathogenesis of myocardium loss and/or apoptosis. The positive results of anti-myocardial antibody test (M2 and MHC) revealed autoimmune mechanism may be involved in the pathogenesis of ARVC. In addition, ARVC should not be regarded as the disease only confined within right ventricle. Corrado et al reported that macroscopic or histologic involvement of the left ventricle was found in 76% of hearts with ARVC [9]. By autopsy of fifty ARVC cases, it is suggested that ventricular distribution is typically biventricular and the most common locations of left ventricular involvement are the posterolateral walls in a subepicardial distribution [17]. Our pathology confirmed the involvement of left ventricle and interventricular septum. Importantly, our case presented heart failure and pathologically right ventricular enlargement and replacement of myocardium by diffuse fibrofatty tissues as well as massive small lymphocytic infiltration. However, the patient has no tachyarrythmias that was defined as characterization of ARVC [1].

Transplantation indications for ARVC have not been defined. In some refractory congestive heart failure, cardiac transplantation is the only therapeutic option [18-20]. Our seven years follow-up has greatly proved the effectiveness of the heart transplantation for ARVC patient.

In conclusion, ARVC without arrhythmias is rare and easily misdiagnosed. Cardiac MRI has become a non-invasive imaging tool in diagnosis of ARVC with high sensitivity and specificity. Inflammatory process may be involved in the mechanism of ARVC.

### Consent

Written informed consent was obtained from the patient for publication of this Case Report and any accompanying images. A copy of written consent is available for review by the Editor-in-Chief of this journal.

## Competing interests

The authors declare that they have no competing interests.

## Authors’ contributions

JW collected the patient’s clinical information and wrote this manuscript. LX conducted the MRI interpretation and drafted the related parts in the article. XC conducted the pathology interpretation and drafted the related parts in the article. JT and DWW revised the article and were in charge of the patient. All authors read and approved the final manuscript.
